# Primary intraosseous carcinoma of the mandible: A report of two cases

**DOI:** 10.4103/0973-029X.72504

**Published:** 2010

**Authors:** Shamindra Sengupta, Hitesh Vij, Ruchieka Vij

**Affiliations:** *Department of Oral Pathology, Institute of Dental Studies and Technologies, Kadrabad, NH-58, Modinagar, District Ghaziabad, Uttar Pradesh, India*

**Keywords:** Carcinoma, jaw neoplasms, malignant neoplasms, primary intraosseous carcinoma, squamous cell carcinoma

## Abstract

Primary intraosseous carcinoma arising as a *de novo* lesion is a unique and rare carcinoma affecting the jaws, especially at younger ages. Two case reports, a 26-year-old Indian female with primary intraosseous squamous cell carcinoma and a 16-year-old Indian male with intraosseous carcinoma arising in an odontogenic cyst, both within the body of the mandible, are presented here.

## INTRODUCTION

Primary intraosseous carcinoma (PIOC) is a unique tumor[1] exclusive to the jaws. The tumor was first described by Loos[2] in 1913 as central epidermoid carcinoma of the jaws and, since then, it has been referred to by a variety of names such as primary carcinoma of the mandible,[3] primary epithelial tumor of the jaw,[4] intraalveolar carcinoma of the jaw,[5] primary intraalveolar epidermoid carcinoma,[6] primary intraosseous carcinoma,[7] primary intraalveolar squamous cell carcinoma (SCC) of the mandible,[8] malignant primary intraosseous carcinoma[9] and central SCC of the mandible.[10] The World Health Organization (WHO)[4] has published criteria to differentiate PIOC from other primary carcinomas and metastatic SCC of the jaws. Additional criteria for accepting a lesion as PIOC have been suggested by Suei *et al*.[5] The clinical and radiological features have been extensively discussed in the literature.[1,3,6,7] The usual histological features of PIOC resemble non-keratinizing SCC with low-to-high mitotic activity.[10] An alveolar or plexiform pattern with peripheral palisading has been reported in a few cases,[1] and sheets of pleomorphic round or oval cells with few mitotic figures have also been observed.[11] This article presents two cases of PIOC with a discussion of the current perspectives.

## CASE REPORT

### Case 1

**Figure 1:**
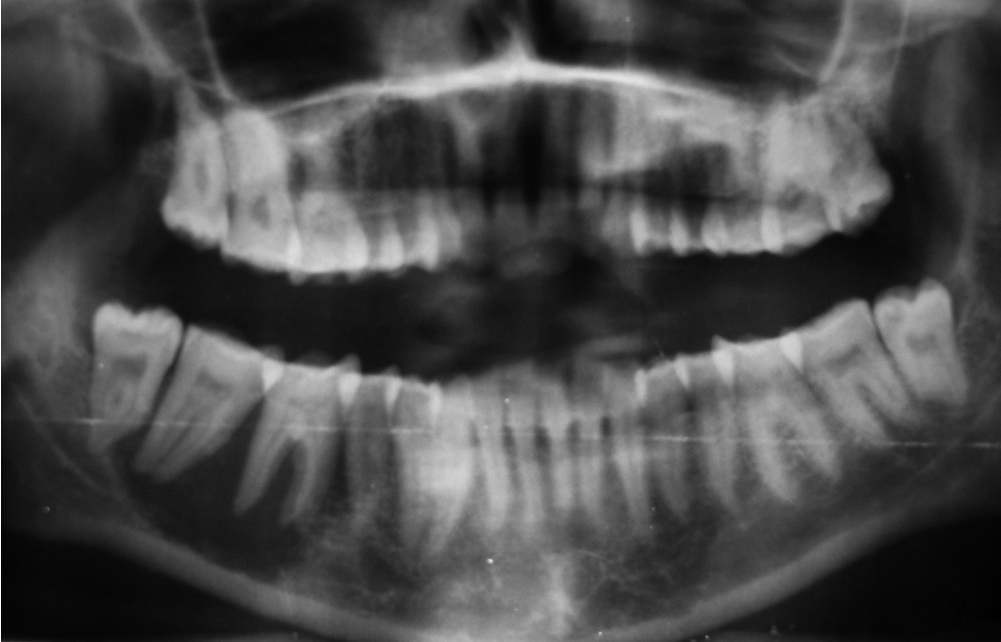
Case 1: Orthopantomogram showing extent of radiolucent lesion in the body of the right mandible

A 26-year-old Indian female reported to the clinics with a complaint of a diffuse dull ache in the molar region of the right mandible since 1 month. Examination revealed no abnormalities other than mobility, of 46, 47, 48 without displacement. No ulceration of the mucosa was observed and there was no initial connection with the oral mucosa or overlying skin. Dento-gingival margins of the mobile teeth were found to be intact, with no periodontal breakdown or pocket formation. An orthopantomogram showed a destructive unilocular radiolucent lesion with diffuse non-corticated margins affecting the body of the mandible on the right side, with the lower border of the mandible being intact [Figure 1]. Attempts to aspirate the lesion produced no cystic fluid. A clinical diagnosis of odontogenic keratocyst was made and the lesion was surgically curetted along with extraction of 45, 46, 47 and 48 under general anesthesia. Healing was uneventful.

### Case 2

**Figure 2:**
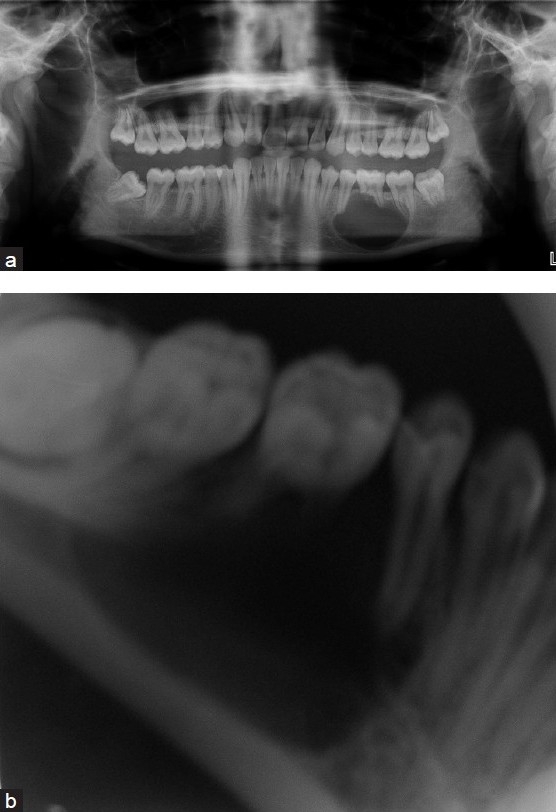
Case 2: (a) Orthopantomogram showing extent of radiolucent lesion in the body of the left mandible. (b) Extraoral lateral oblique view radiograph showing the extent of the root resorption

A 16-year-old Indian male reported to the clinics with a complaint of a mild pain and swelling in the molar region of the left mandible since 2 weeks. On examination, no intraoral ulceration or periodontal pathology was found in the area of the swelling. Radiographic examination showed a unilocular radiolucent lesion with well-defined margins along with root resorption of 36 [Figure 2a and b]. No cystic fluid could be aspirated. Based on clinical and radiological features, a provisional diagnosis of odontogenic keratocyst was made and the lesion was surgically curetted along with extraction of 35, 36 and 37 under general anesthesia.

**Figure 3:**
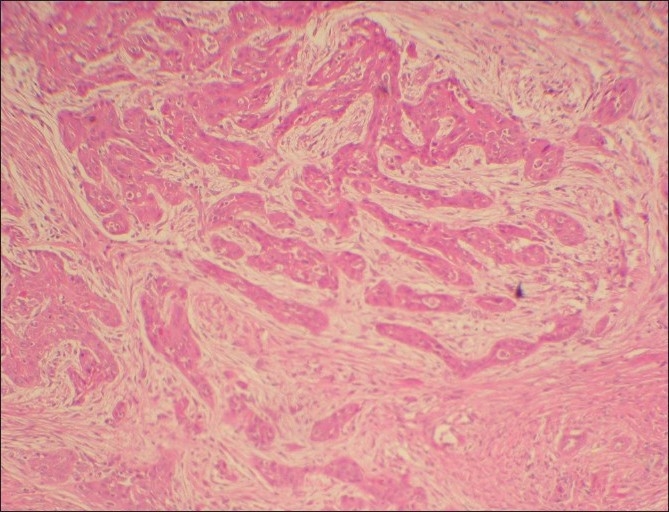
Case 1: Low-power photomicrograph showing islands of infiltrative neoplastic epithelium (H and E, ×100)

**Figure 4:**
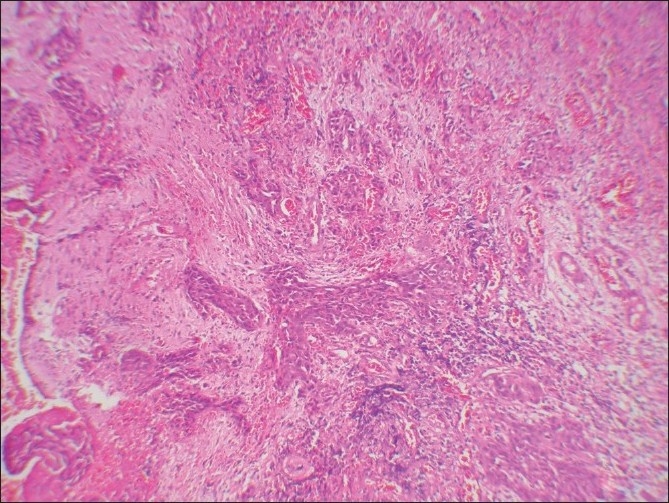
Case 1: Low-power photomicrograph showing neoplastic epithelium with vascular proliferation (H and E, ×100)

**Figure 5:**
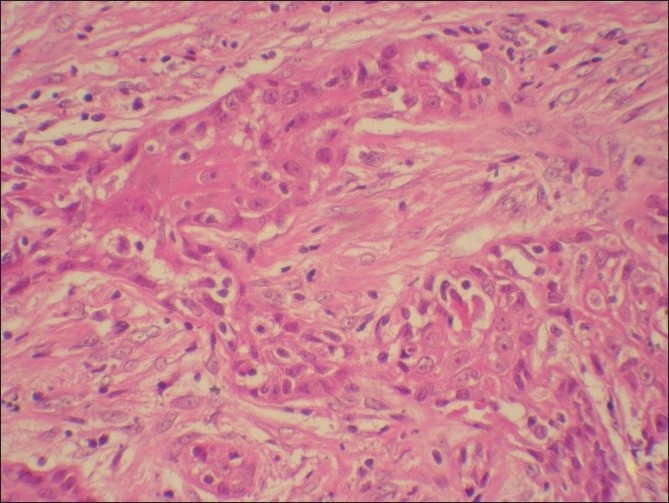
Case 1: High-power view of field in Figure 3 (H and E, ×400)

**Figure 6:**
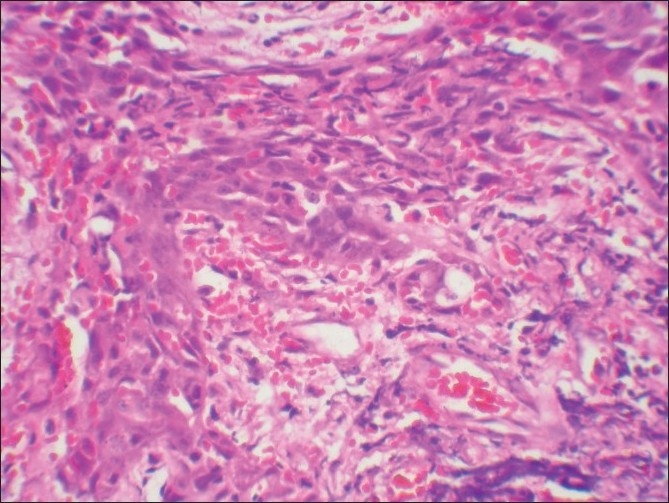
Case 1: High-power view of field in Figure 4 (H and E, ×400)

**Figure 7:**
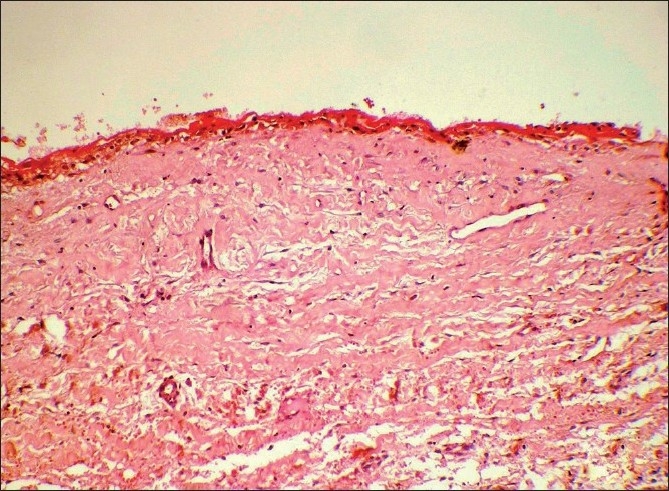
Case 2: Low-power photomicrograph showing cystic lining and islands of neoplastic epithelium in deeper tissue (H and E, ×40)

**Figure 8:**
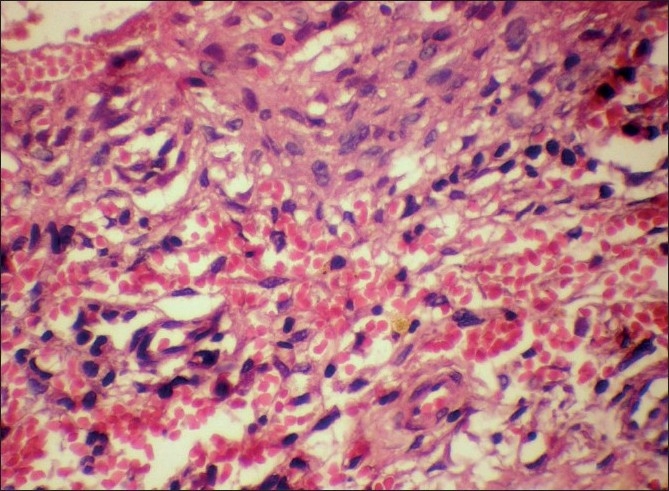
Case 2: High-power photomicrograph showing anaplastic changes in the cystic epithelium (H and E, ×400)

Microscopic examination of both the cases revealed morphologically altered epithelial cells invading into the connective tissue with scattered anaplastic epithelial cells as well as epithelial island formation. Malignant features, including cellular and nuclear pleomorphism and hyperchromatism, were prominent. A few areas showed the presence of intercellular bridges and cytoplasmic accumulation of keratin, characteristics indicative of squamous origin [Figures 3–8]. Some eosinophilic osteoid formation was observed, although it could be considered to be dentinoid owing to its close association with odontogenic epithelial cells.[11] No tissue material resembling an ameloblastoma or an odontogenic keratocyst lining was observed, even with serial sections. A histopathological diagnosis of primary intraosseous carcinoma of the mandible was made. Both the patients were subsequently referred to the Rajiv Gandhi Cancer Hospital, New Delhi, India, for treatment and were lost to follow-up.

## DISCUSSION

The WHO defines PIOC of the jaw as a “squamous cell carcinoma arising within the jaw, having no initial connection with the oral mucosa and presumably developing from residues of the odontogenic epithelium.” PIOC was subclassified into carcinoma arising *de novo*, carcinomas arising from ameloblastoma and carcinomas arising from odontogenic cyst.[4] It appeared logical to also include intraosseous mucoepidermoid carcinomas as a fourth type of PIOC.[6] The current classification is as follows:[10]

Type 1: PIOC ex-odontogenic cyst

Type 2A: Malignant ameloblastoma

Type 2B: Ameloblastic carcinoma arising *de novo*, ex-ameloblastoma or ex-odontogenic cyst

Type 3: PIOC arising *de novo*: (a) Keratinizing type: (b) Non-keratinizing type

Type 4: Intraosseous mucoepidermoid carcinoma

The WHO’s[1] histological classification mentions that PIOCs can develop from odontogenic epithelium. Lucas[8] presumed that this carcinoma could arise from odontogenic rests or from epithelium trapped within deeper structures during fusion of facial process. However, investigators now believe that embryonic tissues, except for the epithelial component of the incisive canal, have little or no pathogenic significance in the development of jaw cyst or tumors.[9] For a carcinoma to develop, presence of epithelium is a must, and it can include remnants of dental lamina, remnants of Hertwig’s epithelial root sheath, remnants of enamel organ, odontogenic tumors or odontogenic cyst lining, entrapped epithelium during fusion of facial process and salivary gland inclusions.[6] The present tumors were not associated with any embedded or missing teeth. Cystic component was observed in one case. The tumors were diagnosed to be well-differentiated SCC presumably arising *de novo* from the cell rests of Malassez or from an odontogenic cyst.

The pooled analysis of the world literature of PIOC[10] reveals that the mean age of the patients of PIOC is 52.3 years, with the age ranging from 4 years to 81 years, with 65% of the patients being in the sixth or seventh decades of life.

The tumor affects men more often than women, with a male:female ratio ranging from 1.5 to 3.5:1.[1,7,10,12] Majority of the cases arise in the posterior mandible where remnants of the dental lamina are most likely to be the source of epithelium. Thomas *et al*.[10] have reported that 77.14% cases occur in the posterior mandible. Only a few occur in midline (anterior mandible), indicating that some lesions may have arisen from epithelial remnants in line of fusion of facial processes.

Although the clinical features of PIOCs are non-specific, Thomas *et al*.[10] have reported that principal manifestations of *de novo* PIOC cases were sensory disturbances like paraesthesia and numbness, mimicking facial neurological problems.

The histologic features of PIOC are not pathognomonic and diagnosis is often difficult. This is because the histologic variation of the odontogenic epithelial component in odontogenic cysts and tumors can be quite confusing. The histology must be that of SCC. Initially, Shear[3] said that PIOC has absence of keratinizaion. But, this was contradicted by Elzay,[1] who pointed out that it is not necessary that PIOCs are non-keratinized SCC. Rather, they can also be keratinized. Thomas *et al*.[10] reported a majority of solid PIOC to be keratinizing and that there was no component of cystic or other odontogenic tumors in the cases.

Shear[3] pointed out that the histological appearance of PIOC is fairly characteristic, with features suggesting the origin of these tumors from the odontogenic epithelium. These can have an alveolar or, sometimes, a plexiform pattern, with palisading of the peripheral cells. It is important to recognize that all these features are suggestive of ameloblastoma. PIOCs originating from odontogenic cysts will show stratified squamous epithelium showing neoplasia as well as cystic lining. The histological picture is thus not very pathognomonic and the microscopy will include all the lesions that produce squamous odontogenic epithelium. These include acanthomatous ameloblastoma, squamous odontogenic tumor and benign and malignant salivary gland tumors that present with squamous metaplasia.

Diagnostic criteria were mainly established by the work of To ENW *et al*.[7] and Suei *et al*.[5] For the diagnosis of a tumor as PIOC, the existence of a primary tumor in another site must be ruled out. The possibility that the lesion is a metastatic should be considered but must be ruled out by obtaining a careful history and thorough physical examination along with radiographic skeletal survey, whole body computed tomography scan or ultrasonography and routine hematological and biochemical studies. A negative chest radiograph is necessary with a post-treatment observation period of 6 months. In the event of an early death, an autopsy reporting no evidence of another primary site is mandatory.

The mucosal condition is also very important to differentiate the PIOC from surface SCC. Suei *et al*.[5] proposed three criteria for the diagnosis of PIOC:

1. To differentiate it from SCC of surface mucosal origin, no ulcer formation must be present on the overlying mucosa except that due to causes such as trauma or tooth extraction.
2. To rule out the possibility of another odontogenic carcinoma, serial sections of histological specimens must demonstrate SCC without cystic component or other odontogenic tumor cells.
3. To rule out distant primary tumor, the chest radiograph must be clear at the time of diagnosis and for a follow-up period of more than 6 months.

Treatment for PIOC is principally wide local resection and radiotherapy and chemotherapy may be performed as adjunctive treatments. Its prognosis is relatively poor and the 5-year survival rate ranges from 30% to 40%,[7,10] with a mean survival rate of 40.6 months if treated by radical surgery with radiotherapy.[13] PIOC is occasionally, but not always, associated with metastasis to the regional lymph nodes.[5] In both the cases, no regional lymph node enlargement was observed at the initial examination. However, ipsilateral lymphadenopathy was observed 1 week after the tumors were curetted. In these cases, the patient underwent hemimandibulectomy with radical neck dissection.

In conclusion, the purpose of reporting these cases is to add to the existing database about this rare tumor, which will further help in an establishment of theories about the origin and biological behavior of these truly uncommon neoplasms.
